# An in vivo evaluation of the change in the pulpal oxygen saturation after administration of preoperative anxiolytics and local anesthesia

**DOI:** 10.15171/joddd.2016.005

**Published:** 2016-03-16

**Authors:** Krishna P. Shetty, Sarvepalli V. Satish, Krishnarao Kilaru, Kalyana Chakravarthi Ponangi, Alexander M. Luke, Srisha Neshangi

**Affiliations:** ^1^Professor and Head, Department of Conservative Dentistry and Endodontics, Navodaya Dental College and Hospital, Raichur, Karnataka, India; ^2^Professor, Department of Conservative Dentistry and Endodontics, Navodaya Dental College and Hospital, Raichur, Karnataka, India; ^3^Endodontics. Bneid Al–Gar Dental Centre, Ministry of Health. Kuwait; ^4^Postgraduate Student, Department of Conservative Dentistry and Endodontics, Navodaya Dental College and Hospital, Raichur, Karnataka. India; ^5^Senior Lecturer, College of Dentistry, Ajman, United Arab Emirates; ^6^Consultant Dental Surgeon, Hyderabad, Telangana. India

**Keywords:** Adjuvants, anesthesia, anxiolytic effect, local anesthesia, midazolam

## Abstract

***Background.*** Given the influence of systemic blood pressure on pulpal blood flow, anxiolytics prescribed may alter the pulpal blood flow along with the local anesthetic solution containing a vasoconstrictor. This study evaluated the impact of preoperative anxiolytics and vasoconstrictors in local anesthetic agents on pulpal oxygen saturation.

***Methods.*** Thirty anxious young healthy individuals with a mean age of 24 years were randomly selected using the Corah’s Dental Anxiety Scale (DAS). After checking the vital signs the initial pulpal oxygen saturation (initial SpO2) was measured using a pulse oximeter. Oral midzolam was administered at a dose of 7.5 mg. After 30 min, the vital signs were monitored and the pulpal oxygen saturation (anxiolytic SpO2) was measured. A total of 1.5 mL of 2% lidocaine with 1:200000 epinephrine was administered as buccal infiltration anesthesia and 10 min the final pulpal oxygen saturation (L.A SpO2) was measured.

***Results.*** The mean initial (SpO2) was 96.37% which significantly decreased to 90.76% (SpO2) after the administration of the anxiolytic agent. This drop was later accentuated to 85.17% (SpO2) after administration of local anesthetic solution. Statistical significance was set at P<0.0001.

***Conclusion.*** High concentrations of irritants may permeate dentin due to a considerable decrease in the pulpal blood flow from crown or cavity preparation. Therefore, maintaining optimal blood flow during restorative procedures may prevent pulpal injury.

## Introduction

The pulp tissue is highly vascular with rich capillary network surrounded by connective tissue in rigid dentinal walls. The blood volume sums up to 3% of the wet weight.^1^ The resting pulpal blood flow is four times that of the resting skeletal muscle, averaging 0.15-0.17 mL/min/g.^2^ The endothelium of the dental pulp capillaries is continuous throughout, except in the odontoblastic region.^3^This suggests the higher rate of metabolic activity and transcapillary fluid exchange in this region.

Vasoconstrictor-containing local anesthetics reduce the pulpal blood flow to a great extent.^4,5^ The action of adrenaline on the alpha receptors of blood vessels resulted in a sharp decline in pulpal blood flow after anesthetic administration causing vasoconstriction.^6^Since local anesthetics are commonly employed in restorative procedures, it would be evident that the pulpal blood flow may be compromised, resulting in tissue injury.^7^

The influence of systemic blood pressure on the regulation of pulpal blood flow is greater than that of local vasoconstriction.^8^

Anxiolytics do cause a change in the electrolyte balance, thereby eliciting a decrease in systemic blood pressure,^9^ which might alter the pulpal blood flow. Changes in the systemic perfusion pressure alter the pulpal blood flow under sedation due to the lower resting level of sympathetic activity and autoregulation.^10^Therefore, the administration of anxiolytics might lower the pulpal blood flow.

Hence the main objective of this study was to evaluate the impact of preoperative anxiolytic medications and vasoconstrictor-containing local anesthetics on pulpal oxygen saturation.

## Methods

Informed consent was obtained from the human subjects who participated in this experimental investigation after the risks and benefits of participation were described to the subjects or patients recruited and the Institutional Review Board approved the protocol. This study conformed to the Helsinki Declaration of 1975, adhering to the guidelines of the American Society of Anesthesiologists (ASA).

Thirty anxious subjects were randomly selected using the Corah’s Dental Anxiety Scale (DAS) from the Outpatient Department, Navodaya Dental College and Hospital, Raichur.

### Inclusion criterion

Healthy young individuals with a mean age of 24 years, weighing 60-75 kilograms were included in this study.

### Exclusion criteria 

Patients with cardiovascular instability, chronic renal failure, open-angle glaucoma, respiratory disease, myasthenia gravis, history of drug or alcohol abuse and hepatic impairment were excluded from the study.

Fully erupted maxillary central incisors devoid of caries, restorations, developmental defects, mobility, root resorption and any symptoms of pain were selected.

The patient was asked to sit on the dental chair and after checking the vital signs (pulse, respiratory rate and blood pressure), the initial pulpal oxygen saturation (initial SpO_2_) was measured using the Pediatric SaO_2_ Sensor Two Piece (Nellcor, Covidien, Dublin, Ireland) and Handheld Pulse Oximeter. (G1B, General meditech, China) by using the rubber dam clamp as the base for the sensor design. This method was previously employed by Noblette et al.^10^ Midzolam (7.5 mg) (Mezolam, Neon Laboratories, Mumbai, India) was was administered orally. After an interval of 30 min, the vital signs were monitored and the pulpal oxygen saturation (anxiolytic SpO_2_) was measured. A total of 1.5 mL of 2% lidocaine with 1:200000 epinephrine (Xynova 2%, Triokaa Laboratories, Dehradoon, India) was administered as the buccal infiltration anesthesia and after 10 min the final pulpal oxygen saturation (L.A SpO_2_) was measured. Three pulpal oxygen saturation (SpO_2_) values were registered at each interval within 30 s to 3 min of monitoring and the mean was taken as the final reading**.**

Data were expressed in terms of mean and SD. Repeated-measures ANOVA was used for analysis of data, followed by pairwise comparisons with Tukey test. A two-tailed p-value less than 0.05 was considered as significant.

## Results

**Figure 1.**
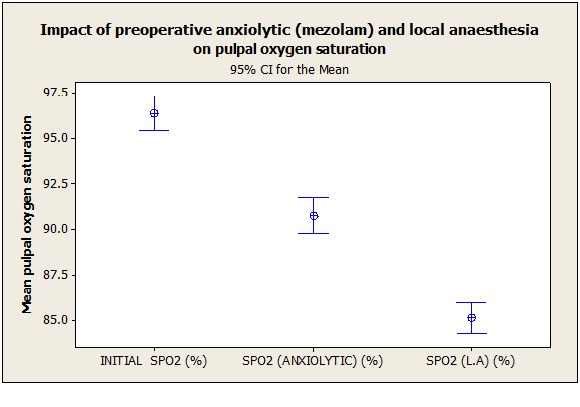
Mean pulpal oxygen saturation levels. Initial, anxiolytic and local anesthetic SpO_2_

The mean initial (SpO_2_) was 96.37%, which significantly dropped to 90.76% (SpO_2_) after the administration of the anxiolytic agent. This drop was later accentuated to 85.17% (SpO_2_) on the administration of local anesthetic solution (Table 1 & 2; Figure 1). A p-value less than 0.0001 was considered to be significant.

**Table 1:** Comparison of initial, anxiolytic and local anesthetic SpO^2^

**Group**	**Mean**	**SE**	**F-value**	**P-value**	**Tukey test**	**Mean difference**	**95% CI of difference**
**Initial** SpO_2_** (%)**	96.37	0.46	1396	P<0.0001	I vs II, P<0.001	5.60	5.09 – 6.11
SpO_2_** (Anxiolytic) (%)**	90.76	0.47			I vs III, P<0.001	11.20	10.69 – 11.71
SpO_2_** (L.A) (%)**	85.17	0.41			II vs III, P<0.001	5.6	5.09 – 6.11

## Discussion

Administration of an anxiolytic medication significantly decreased the pulpal oxygen saturation, which further decreased by the administration of local anesthesia with epinephrine.

The arteries of the dental pulp branch into a capillary network and exit the apical foramen as venules. Dental pulp, due to its enclosure in dentin, has lower compliance, higher blood flow and volume. The interstitial fluid pressure (IFP) and colloidal osmotic pressure of dental pulpal tissue are high in contrast to the net driving blood pressure.^11^

Catecholamine application reduces the dental pulpal circulation within a few minutes even before the inhibition of nerve activity.^5^

Deficient pulpal blood flow may dampen the dentinal tubular fluid flow outward. This in turn may potentiate the risk of the penetration of noxious stimuli through the dentinal tubules.^11^

Higher blood flow in the dental pulpal tissues, during restorative procedures tends to dissipate heat and carry away bacterial toxins.^12^Vasoconstrictor-containing local anesthetics do cause a significant decrease in the pulpal blood flow.^4,6^ Our study was consistent with the above findings. The immune response in an inflamed pulp may be compromised due to deficits in the blood flow, leading to detrimental effects to the tissue.^12^

Sedation is employed commonly as an adjunct to local anesthesia for apprehensive patients.^13-15^ Midazolam commonly used in pediatric dentistry was the drug of choice in this study because of its wide toxic/therapeutic ratio and safety margin and absence of the prolongation of the period of sedation like benzodiazepines. It is rapidly absorbed, produces its peak effect in a relatively short time of about 30 min, and has a short half-life of about 1.75 hours.^16^

The influence of systemic blood pressure on the regulation of pulpal blood flow is greater than that of local vasoconstriction or vasodilation.^8^ Anxiolytics tend to reduce the heart rate and alter the electrolytic balance and the plasma osmolality, thereby reducing the systemic blood pressure. In this study, there was a 10% reduction in the mean systolic blood pressure as shown in Table 2. Therefore, the change in the systemic blood pressure brought about by the anxiolytic also influenced the pulpal blood flow.

**Table 2:** Differential mean systolic blood pressure

**Total number of cases chosen**	40
**Number of cases with drop in systolic blood pressure**	27
**Initial mean systolic blood pressure (mm Hg)**	135.75
**Post-anxiolytic systemic blood pressure (mm Hg)**	122.17
**Difference in the blood pressure (mm Hg)**	13.57
**Percentage change in the mean systolic blood pressure (%)**	10

In this study there was a fall in the pulpal blood flow after the administration of the anxiolytic, which was accentuated after the administration of the local anesthetic, with possible detrimental effects on the pulpal tissue. None of the previous studies have checked for the pulpal oxygen saturation after anxiolytic administration.

Pulse oximetry is a non-invasive medical monitoring methodology employed to determine the blood oxygen saturation and pulse rate readings in various tissues.^17-20^Pulse oximetry is also used to monitor the oxygen saturation of patients on intravenous anesthesia.^21^ This technique operates on the principles of Beer's law, which states that the absorption of light by a solute depends on its concentration at a given wavelength.^22^

Schmitt et al^22^ in an in vitro tooth model found that pulse oximetry effectively determined the oxygen saturation. Noblett et al^11^accurately determined the pulpal oxygen saturation of a tooth model by employing a rubber dam clamp to function as a base for the sensor design.

The conformation of the sensor to the size, shape and anatomic contours of the tooth plays a critical role in determining the pulpal oxygen saturation. This was the reason for choosing Nellcor pediatric Sensor (Two Piece).

## Conclusion

The rate of clearance of toxins diffusing across the dentinal tubules by the pulpal blood flow influences their concentration in the pulp. The use of a anxiolytic agent as an adjunct to local anesthetic compromised the pulpal blood flow. This compromised pulpal blood flow could result in higher permeation of toxins through the dentin during restorative procedures. Thus, taking these considerations into account in restorative procedures, an attempt should be made to maintain optimal pulpal blood flow and cautiously prescribe anxiolytics. Dental health professionals must exercise caution with the use of anxiolytics. Anxiolytics may be administered only as a last resort to avoid possible pulpal injury.

## Acknowledgment

The authors do not acknowledge an individual or agency.

## Author’s contributions

This study was designed by KPS, SVS, SN and PKC. The experimental methodology was formulated by PKC, KK, SN and KPS. The experiments were conducted by SN, AML, KPS, KK and SVS. The statistical analysis and the data interpretation were carried out by KPS and KCP. The literature review was carried out KCP, SN and AML. The manuscript was drafted by KCP, KK, SN, KCP and SVS. KCP, SN, SVS and KPS participated in the revision of the manuscript.

## Funding

This study was collectively funded by all the authors.

## Ethics approval

Informed consent of the human subjects who participated in the experimental investigation was obtained after the risks and benefits of participation were described to the subjects or patients recruited and the Institutional Review Board approved the protocol. This study conformed to the Helsinki Declaration of 1975 and adhered to the guidelines by the American Society of Anesthesiologists (ASA).
